# Estimating the net value of treating hepatitis C virus using sofosbuvir-velpatasvir in India

**DOI:** 10.1371/journal.pone.0252764

**Published:** 2021-07-22

**Authors:** David E. Bloom, Alexander Khoury, V. Srinivasan

**Affiliations:** 1 Department of Global Health and Population, Harvard T.H. Chan School of Public Health, Boston, Massachusetts, United States of America; 2 Stanford Graduate School of Business, Stanford, California, United States of America; PLOS, UNITED KINGDOM

## Abstract

Recently developed direct-acting antiviral (DAA) treatments for hepatitis C virus (HCV) have been groundbreaking for their high efficacy across disease genotypes and lack of severe side effects. This study uses a cost-of-illness (COI) approach to estimate the net value conferred by this class of drugs using the cost and efficacy of one of these novel drug combinations, sofosbuvir and velpatasvir (SOF/VEL), recently licensed for generic manufacture in India. This study considers COI of lifetime earnings lost by patients and potential secondarily infected individuals due to disability and premature death from HCV infection. Expected net benefits of treatment are substantial for non-cirrhotic (NC) and compensated cirrhotic (CC) patients (ranging from 5,98,003 INR for NC women to 1,05,25,504 INR for CC men). Increased earnings are not sufficient to fully offset cost of treatment for decompensated cirrhotic individuals but treatment may still be justified on the basis of the intrinsic value of health improvements and other treatment benefits.

## I. Introduction

Hepatitis C virus (HCV) is a major global health problem affecting between 120 million and 200 million people worldwide [1]. These infections impose a heavy toll of mortality: the World Health Organization (WHO) estimates that roughly 399,000 people die from HCV each year [2], while other estimates place this figure at more than 1 million people [1]. This mortality is accompanied by a substantial morbidity burden, primarily associated with liver cirrhosis and its complications (including liver failure and hepatocellular carcinoma). These sequelae eventually develop in 10–20% of patients, typically after 20 to 30 years of asymptomatic infection [3].

India holds a substantial portion of the global HCV burden. The number of HCV carriers in India is estimated to be between 3 and 9 million people, with localized prevalence of HCV antibodies reaching as high as 5.2% in some studies [4]. While the incidence of new HCV cases in India is believed to have peaked in 2001, the annual number of new infections remains high, with an estimated 287,920 new cases occurring in 2013. Expert consensus suggests that the primary route of transmission in India is through unsafe medical injection practices, although blood transfusions and intravenous drug use are also important transmission channels [5].

### Promise offered by newly available direct-acting antivirals (DAAs)

For decades, HCV infection was a broadly known, burdensome illness without effective and well-tolerated treatment options. Previously, pegylated interferon (PEG-INF) or non-pegylated interferon delivered in combination with ribavirin (RBV) were the most common HCV treatments. However, the overall efficacy of this treatment course in achieving sustained virological response (SVR) is only between 47% and 63% [6,7]. Interferon ribavirin combination therapy also regularly induces severe side effects, which include hematologic abnormalities, neuropsychiatric symptoms, flu-like symptoms, and fatigue. Because of the severity of these side effects, 10–14% of participants in clinical trials had to discontinue treatment prematurely [6,8]. The low efficacy rates and intolerability of the treatment for individuals with sufficiently advanced cirrhosis (Child–Pugh class B or C) render patients in this population ineligible for interferon therapy. This is particularly relevant in India, where only 45% of patients are diagnosed early enough to be eligible [9].

However, several new treatments developed in recent years, known as direct-acting antivirals (DAAs), have greatly improved the curability of HCV infection. These drugs, which include sofosbuvir, simeprevir, and daclatasvir, feature much higher rates of virological clearance and far better tolerability than previous treatment options [10]. The new DAAs have also been particularly groundbreaking for patients with late-stage liver disease because of their ability to treat HCV effectively without the use of interferon therapy [11]. They can also be used safely in combination with one another, and several of these oral, IFN-free combinations have achieved SVR in 90% of patients after just 12 weeks of treatment. Another advantage of some of these DAA combinations is that they are pangenotypic, meaning they have high rates of efficacy against all six globally prevalent HCV genotypes [12]. This is in contrast to older treatment options, which had rates of efficacy that varied widely by genotype, necessitating that individuals be tested beforehand to determine which drug or combination was appropriate, with some genotypes having no highly effective options [6].

### Assessing the value of SOF/VEL

One of these novel drug combinations, sofosbuvir (400mg) and velpatasvir (100mg) (SOF/VEL) is assessed as representative of the incremental value offered by the new class of DAAs. This study focuses on SOF/VEL specifically because a Nielsen survey of physicians indicated SOF/VEL to be the preferred treatment option of all the DAAs available at the time of the study. This single-tablet regimen, was recently licensed for generic manufacture in India. The combination is particularly valuable for having above 95% efficacy for each disease genotype [13,14], and the current price of a full 12-week course of treatment is 52,500 INR, (or US$ 819) [15]. [Figures throughout the paper assume a conversion rate of 64.07 rupees per US$ [16]]. While this is far cheaper than the brand-name drug, Epclusa, which retails at $73,300 for a 12-week regimen in the United States [17], it constitutes a major expense in India, equal to a nearly half of the country’s gross domestic product (GDP) per capita in 2016 [18]. However, comparing the cost of treatment to the benefits it confers offers another metric for assessing the value of SOF/VEL.

Cost-benefit analysis is particularly useful for determining the net benefits of a given health intervention because it represents costs and outcomes of treatment in comparable monetary units. Generally, costs are fairly easy to monetize, often represented as the sum of the prices of goods and services needed to administer a given intervention. However, many benefits of health interventions, such as decreased morbidity or mortality, are not explicitly traded on economic markets. As such, definitive monetary values for these benefits cannot simply be observed; instead, a deliberate approach must be taken to infer their value from empirical information, normative judgements, or some combination of both.

Among the most versatile and broadly applied methods of determining the value of treatment is the cost-of-illness (COI) approach, which values an intervention according to the estimated economic burden of the disease it treats. By measuring the burden of disease to society, COI studies can play an important role in informing policymakers about the relative importance of different health issues and which interventions offer the most impact for investment. However, those utilizing COI results must pay heed to the methodologies used in each study to ensure they understand exactly which costs these analyses attempt to measure. Failure to do so may lead to misinterpretation of the relative and absolute values of various interventions.

## II. Methodology

The costs examined in COI studies can generally be categorized into three broad groups: direct costs, indirect costs, and intangible costs. Direct costs of illness comprise all expenses individuals, families, health systems, and societies incur as the result of an illness. These include expenditures on diagnosis, treatment, and other healthcare goods and services, as well as nonmedical expenditures necessitated by illness, such as the cost of transportation to medical facilities or relocating the household. Indirect costs include any productivity lost due to illness-related morbidity or mortality [19]. This is not limited to the productivity losses of the primary infected individual but also encompasses downstream effects on individuals or organizations impacted by their illness. Intangible costs include all negative impacts of a disease without a precise market value, such as pain and discomfort.

This study focuses specifically on the indirect costs HCV imposes on market activity and hence is a conservative estimate of the cost of illness. The indirect costs include the disease’s impact on the expected lifetime productivity of primary infected individuals (i.e., HCV-diagnosed patients for whom SOF/VEL treatment is being considered) and any other individuals to whom they may transmit the disease.

Analyses do not consider the direct costs of HCV infection if individuals are not treated with SOF/VEL, as individuals are unlikely to undergo liver transplant or dialysis (both of which are prohibitively expensive for most Indians), or receive other, less-effective HCV medication. Other treatment costs for easing discomfort in the late stages of the disease are assumed to be negligible. Intangible costs are also excluded from analyses because of the ambiguities and uncertainties involved in assigning monetary value to these various outcomes. Excluding these costs allows for results that depend less on arbitrary—and possibly controversial—decisions on value setting.

In accordance with these considerations, the model presented in this paper estimates the individual-level net benefits to market productivity resulting from treatment with SOF/VEL for both primary and secondarily infected individuals. The focus on secondarily infected individuals seeks to capture the benefits associated with the interruption of further HCV transmission, considering the average rate of transmission, the remaining life expectancy of the primarily infected individual, and the expected impact of HCV infection on the lifetime earnings trajectory of each individual to whom the illness would be transmitted. This analysis is particularly valuable for considering economic impacts at the national level from a societal perspective. We contrast our approach to that presented in Aggarwal et al. [20], which determines the cost-effectiveness of DAA treatment for HCV by estimating the improvements in quality-adjusted life years (QALYs) and disability-adjusted life years (DALYs) through DAA treatment as well as future medical costs averted by treatment. They consider treatment to be cost-effective if the incremental cost-effectiveness ratio (ICER) of QALYs preserved is less than three times India’s per capita GDP. While this threshold is widely used to evaluate the cost-efficacy of various medical interventions, it is ultimately an arbitrary value, intended to reflect a typical estimate of the statistical value of life. In comparison to this general rule-of-thumb measure our value estimates refer specifically to the likely impacts of treatment on future productivity as determined using empirical information.

The process for estimating these benefits is divided into four stages: (i) estimating age-earnings trajectories for Indian adults with average health, (ii) calculating risk of HCV-related disability and premature death if not treated, (iii) estimating the value of earnings preserved by curing HCV infection, and (iv) applying the value of curing HCV to estimate the net value conferred by SOF/VEL treatment.

### II.i. Estimating age-earnings trajectories for Indian adults with average health

**Fig 1:**
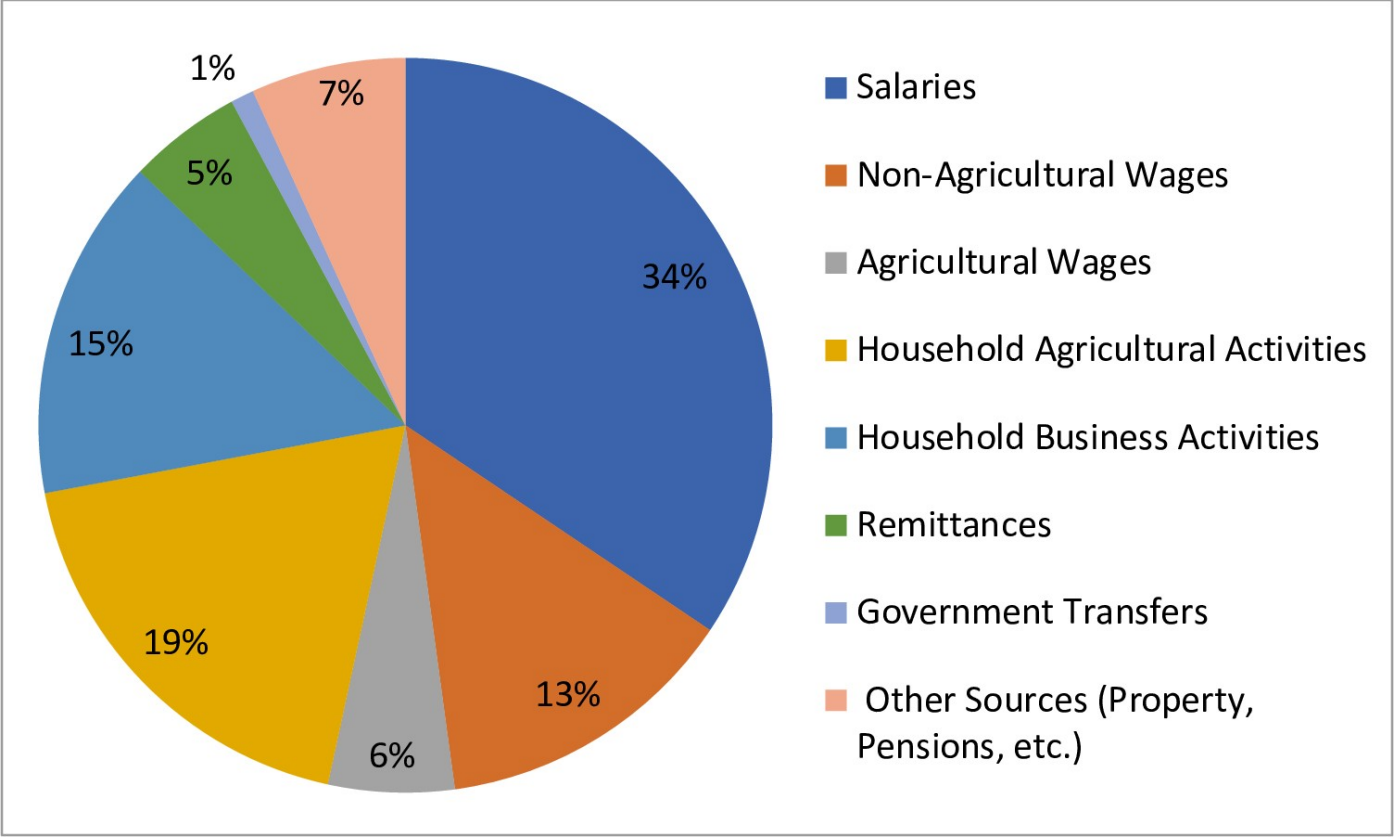
Breakdown of mean household income by source. The analysis separately determines parameters for estimating (a) the likelihood of an individual working and (b) their earnings conditional on working.

The earnings profile and employment status of an Indian adult with average health provide a basis for the expected value of each year of earnings lost due to HCV infection. Throughout this analysis, individuals with average health are used to represent individuals who do not have HCV. These two populations are considered equivalent because HCV-positive Indians make up less than 1.2% of the total population, and therefore have a negligible impact on the population average values. The parameters of these models are estimated using data from the second wave of the Indian Human Development Survey (IHDS) [21]. The IHDS is a nationally representative, longitudinal survey that includes detailed information on household income and demographic characteristics. Data for the survey’s second wave were collected in 2012 and cover 204,569 individuals of all ages in 42,152 households across the India. Table 1 provides basic summary statistics for the wave 2 sample and Table 2 provides basic employment and earnings statistics. Fig 1 breaks down mean household income by source, showing that 87% of the average household’s income comes from salaries, wages, agriculture, and business activities. The summary statistics provided in Tables 1 and 2, and Fig 1 represent unweighted mean values.

**Table 1:** IHDS wave 2 summary statistics.

	Mean/%
***Age***	29.8
***% Female***	50.1
***% Married***	48.3
***Education level***	
*Illiterate*	29.6
*Literate with no formal schooling*	3.2
*Up to 4 years of school*	13.8
*5–9 years of school*	30.4
*Secondary/higher secondary*	15.5
*Some college/bachelor’s degree*	5.6
*Above bachelor’s degree*	1.9
***N = 204*,*569***	

**Table 2:** IHDS wave 2 employment and earnings statistics.

	% Employed	Mean earnings
***Overall***	56.9	52,113
***By sex***		
*Male*	75.7	66,790
*Female*	38.9	25,485
***By age***		
*<15*	6.7	5,231
*15–29*	47.8	36,299
*30–44*	71.4	59,057
*45–59*	68.8	69,083
*60–74*	43.4	36,888
*75+*	16.1	39,856
***% of population working***		
*For wages and salaries*	53.3	52,906
*In household agricultural activities*	29.2	20,939
*In household businesses (nonagricultural)*	8.5	66,043

**Notes:** Earnings and employment statistics are presented for individuals aged 15 and older, except where presented by specific age group. The mean earnings are presented in rupees conditional on participation in remunerative employment, household agricultural activities, or household nonagricultural business activities. Under the *% of population working* section mean earnings refer to mean earnings within the given category, conditional on participation in that activity.

Any individual (age 15 or older) who earned wage or salary income, or who participated in income-generating household enterprise activities (agricultural or nonagricultural) over the last year is considered to be working. The remaining population is considered to be nonworking. A binary indicator of work status (based on the aforementioned criteria) is used as a dependent variable in a probit regression that includes the following control variables: age, squared-age, sex, education level, marital status, number of members of household, and state of residence.

These regressors are chosen because of their standard usage in the labor economics literature and because they are included in both the IHDS data and the Nielsen HCV patient questionnaire conducted in India in fall 2016 (N = 300). (Note that these regressors do not include whether the individual lives in an urban area, has other co-morbidities, or engages in risky behavior. Although these factors are associated with both likelihood of HCV infection and earnings, they were not included in the Nielsen survey.) S1 Table in the appendix in S1 File includes the parameter estimates for this model. The model is estimated using the unweighted sample but applying the cross-sectional weights provided in the IHDS dataset yields similar results.

The individual earnings variable used in the analysis is constructed as the sum of (1) annual earnings from wages and salaries, (2) annual income from agricultural self-employment activities, and (3) annual income from nonagricultural self-employment activities. (If total earnings are negative, which can result from agricultural or business losses, the value of the variable is replaced with 0.) Income from agricultural and nonagricultural self-employment activities is provided at the household level in the IHDS. The following formula is used to estimate each household member’s individual earnings from these agricultural and nonagricultural activities:

$$e=\frac{h}{H}*I$$

where

*e* = annual individual earnings from the self-employment activity,

*h* = hours of labor the individual contributed to the self-employment activity over the last year,

*H* = total hours of labor all household members contributed to the self-employment activity over the last year, and

*I* = total household income from the self-employment activity.

This formula assumes that the productivity of an hour of labor is equal among household members. It also assumes that there is no complementarity or congestion between household members labor (i.e., an individual’s labor has no positive or negative effect the productivity of other household members).

A Poisson model is then used to regress this combined earnings figure against the same independent variables used in the employment regression. In repeated trials of half-sample cross-validation, the Poisson model produced far more accurate earnings predictions than the typically used log-linear model. Furthermore, robust standard error (SE) estimates are used to ensure that the SEs are consistently estimated. For a detailed discussion of the appropriateness of applying the Poisson regression to predict nonnegative variables, refer to Santos Silva & Tenreyro [22] and Wooldridge [23]. S1 Table in the appendix S1 File also includes the parameters estimated by this Poisson regression. Again, these results are generated from the unweighted sample. Weighting the sample does not qualitatively change the results.

The coefficients that these probit and Poisson models generate can be used to estimate the likelihood of working and average earnings conditional on working for an individual with given characteristics and average health status. Multiplying these two predictions yields expected earnings. Multiplying these predictions to yield expected earnings relies on the assumption that employment and earnings conditional on employment are probabilistically independent. This may introduce some bias insofar as employment status may be correlated with potential earnings. However, use of a “seemingly unrelated regression” method would not improve the predictions because the predictor matrixes are identical across both regressions [24].

This study uses separate data from the Nielsen HCV patient survey to assess the sociodemographic characteristics and disease status of individuals *diagnosed* with HCV in India. Table 3 provides basic summary statistics of this survey population. Note that this sample is, on average, much more highly educated than members of the general Indian population (see Table 1). This is unsurprising given that utilization of the allopathic medical system—and thus likelihood of being screened for HCV viremia—is correlated with income in India [25]. It is important to emphasize that, throughout the paper, the value conferred directly to the patient refers to the population diagnosed with HCV+, not the general HCV+ population of India. The productivity-related benefits of treating the average member of the HCV-*diagnosed* population are expected to be much higher than those of treating the average member of the HCV-*infected* population, due to the positive correlation between diagnosis and level of education (and, by inference, productivity). If HCV screening and diagnosis were to become more widespread (perhaps in response to the availability of cheaper, more effective treatments), the characteristics of the diagnosed population would be expected to shift closer to those of the general Indian population. In this case, the results presented in this paper would overestimate the average value conferred to patients to the extent that the current HCV-diagnosed population has higher productivity than the diagnosed population in the future period. By contrast, secondarily infected individuals are assumed to reflect the general Indian population (as represented in the IHDS) and their COI estimates face no risk of bias due to changes in patterns of diagnosis.

**Table 3:** Nielsen patient survey summary statistics.

	Mean/%
***Age***	44.9
***% Female***	37%
***% Married***	86%
***Education level***	
*Illiterate*	0.0%
*Literate with no formal schooling*	0.3%
*Up to 4 years of school*	4.3%
*5–9 years of school*	10.3%
*Secondary/higher secondary*	32.0%
*Some college/bachelor’s degree*	12.3%
*Above bachelor’s degree*	40.6%
***Liver function***	
*Non-cirrhotic*	35%
*Compensated cirrhotic*	34%
*Decompensated cirrhotic*	31%
***N = 300***	

Each independent variable utilized in the IHDS regressions described previously is also present in the Nielsen HCV patient questionnaire. Thus, for any Nielsen respondent it is possible to estimate the earnings of a member of the Indian population with average health status (i.e., not HCV^+^) and the same demographic, social, and household characteristics, as the respondent. Given that invasive medical procedures and blood transfusions are typical routes of transmission in India, comparator individuals would be more likely to have a health condition requiring one of these procedures. As such they would likely have somewhat worse health status on average than typical members of the Indian population, with similar sociodemographic characteristics. This suggests that the remaining life earnings of these counterfactual individuals may be somewhat overestimated.

### II.ii. Calculating risk of HCV-related disability and premature death if not treated

Chronic HCV carriers are typically asymptomatic for many years, with substantial disability usually occurring in the late stages of the disease. The model assumes that individuals experience no disability until they develop decompensated cirrhosis or liver cancer (hepatocellular carcinoma or HCC), corresponding to the assumptions made in Wong et al. [26]. Thus for the purposes of this study “risk of disability” is defined as the probability of an individual being afflicted with decompensated cirrhosis or HCC at a given time (*t*).

Calculations of this risk use age- and sex-specific disease transition rates and HCC mortality from Razavi et al. [27] as well as annual cirrhosis decomposition and death rates from D’Amico [28]. India has very high relative prevalence of HCV genotype 3 [29], which poses greater risk of cirrhosis and HCC incidence than other HCV genotypes [30]. The Razavi et al. [27] disease progression rates derive from U.S. data, where prevalence of genotype 3 is lower. To ensure that the disease progression parameters used in the paper reflect the higher prevalence of the more dangerous genotype 3 in India, a disability risk adjustment factor was calculated based on the relative prevalence of HCV genotypes 1, 2, 3, and 4 in South Asia versus high-income North America, as presented in Messina et al. [29]. (Genotypes 5 and 6 are not considered because the prevalence of these genotypes is very low in both regions; together, genotypes 5 and 6 account for approximately 0.7% of HCV carriers in high-income North America and 0.2% of carriers in South Asia.) These calculations suggest that the annual cirrhosis incidence rate for HCV-positive individuals in India is 1.21 times greater than in North America and the annual incidence rate of HCC is 1.52 times greater. To reflect this higher incidence, transition rates from stage 3 fibrosis to cirrhosis are multiplied by 1.21 and the transition rates from cirrhosis to HCC are multiplied by 1.26 (1.21×1.26 = 1.52). Data limitations prohibit the study from accounting for the higher rates of intravenous drug use among HCV-positive individuals in the United States or the higher rates of malnutrition in India, both of which could affect disease progression rates. These biases are assumed to offset one another but the magnitude and direction of their net impact is ambiguous and they may also introduce a heterogeneity bias as described in Kuntz and Goldie [31].

The risk estimation model starts at the time of observation (*t =* 0) with a hypothetical million-person cohort. The members of this initial cohort are homogeneous with respect to age, sex, and stage of disease progression (either fibrosis, stage 0–3, compensated cirrhosis, or decompensated cirrhosis). No individuals in the patient dataset was recorded as having HCC at the time of interview. As such, HCC is not listed as an *initial* disease state, but individuals may still transition into this state from compensated cirrhosis. The possibility of transition from decompensated cirrhosis to HCC is ignored because both are treated as equivalent states of disability in terms of labor productivity (i.e., individuals are assumed to be unable to work in either case). In each successive year (*t* = 1, *t* = 2, *t* = 3…) the number of individuals advancing to the next disease stage is calculated using the previously described adjusted transition rates. The number of individuals dying from decompensated cirrhosis and HCC (henceforth referred to as “liver-related mortality”) is also calculated using the decompensated cirrhosis and HCC death rates provided in D’Amico [28] and Razavi et al. [27]. In addition, normal age- and sex-specific mortality rates are used to move a proportional number of individuals from all disease stages into an “other mortality” category, which represents mortality from all non-HCV-related causes. This yields a distribution of individuals across disease stages and predicts the number to have died from liver disease and other causes in each year after observation. The risk of disability *j* years after observation is then calculated by dividing the number of individuals with decompensated cirrhosis or HCC at *t = j* by the size of the initial cohort (1 million individuals). The risk of premature death due to HCV is calculated by summing the likelihoods of liver-related mortality and other mortality and then subtracting the likelihood of death for a normal member of the population of the same age and sex (referred to as “counterfactual mortality”). Note that this approach necessitates the construction of separate cohort distribution tables for each age at observation (0–81), initial disease stage (F0, F1, F2, F3, compensated cirrhosis, and decompensated cirrhosis), and sex (constituting 984 tables in total), because risk of disability and death in a given future year varies with all of these factors.

**Fig 2:**
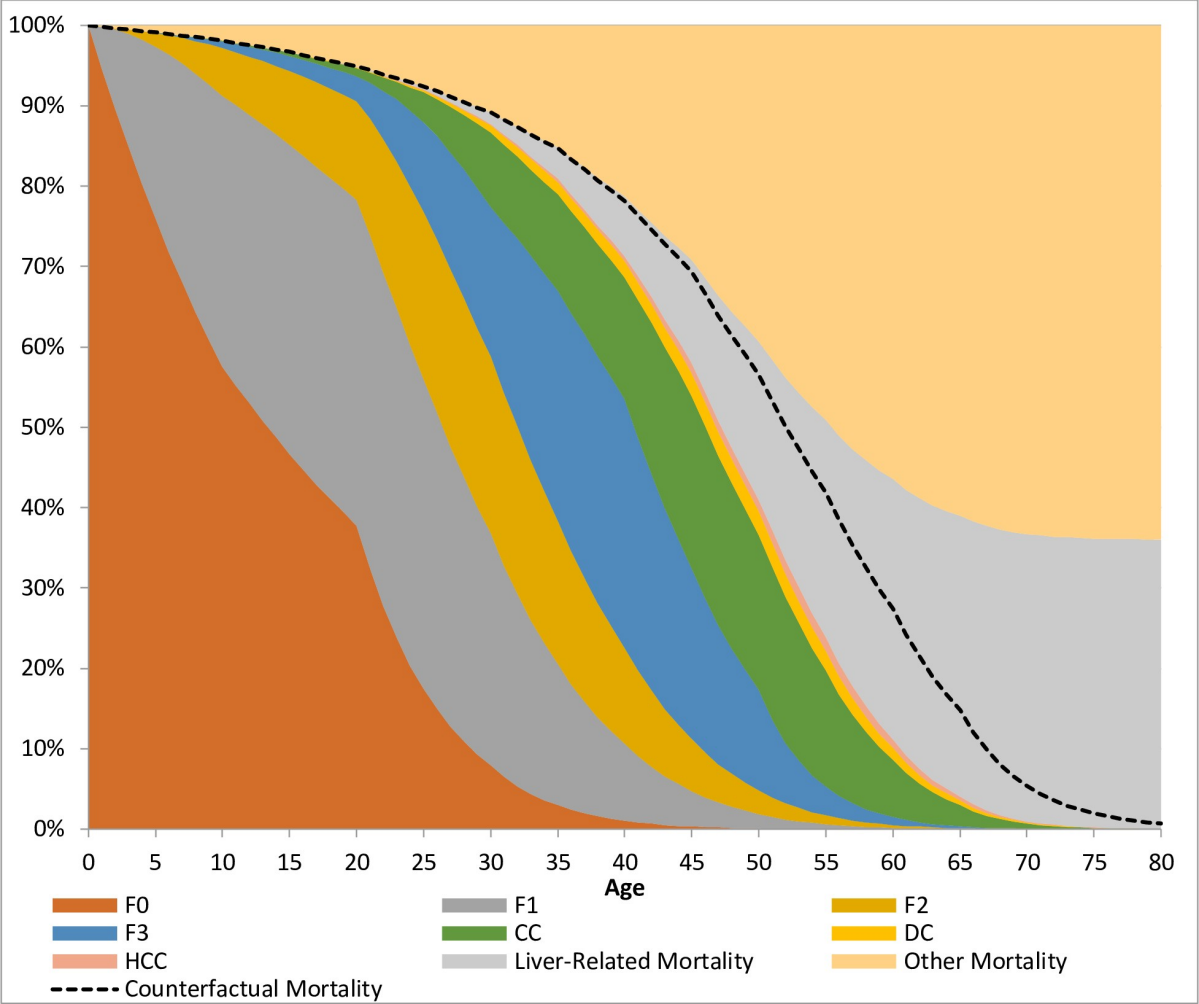
Distribution of disease status by age (HCV+ 20-year-old male cohort starting with F0 fibrosis).

Fig 2 charts one of these 984 tables: the distribution of disease status by future age for a cohort of 20-year-old HCV-positive males with stage 0 fibrosis. The area in pale green (liver-related mortality) below the dotted line (counterfactual mortality) corresponds to the likelihood of premature death due to HCV infection. It is worth noting that the bands representing disability (yellow and pink) are fairly narrow in comparison. This conforms to expectation considering the long time lag from initial infection with HCV to the symptomatic stages of the disease and the high risk of mortality once these stages are reached.

### II.iii. Estimating the value of earnings preserved by curing HCV infection

The value of earnings preserved by curing HCV infection can be divided into four categories: (a) earnings lost to disability (*L*_*dis*_), (b) earnings lost to premature death (*L*_*death*_), and earnings lost by secondarily infected individuals due to (c) disability (*X*_*dis*_) and (d) premature death (*X*_*death*_). This assumes that individuals who are cured of HCV infection are no more likely than the average member of the population to contract HCV a second time. This may introduce an inflationary bias to the estimated value of curing infection insofar as these individuals still have the same underlying health conditions or behaviors that led them to be exposed to infection initially. The total value of earnings preserved by curing HCV infection (*C*) is the sum of these four components:

$$C=L_{dis}+L_{death}+X_{dis}+X_{death}.$$

#### Benefits of averted disability (L_dis_)

For HCV-positive individuals who are non-cirrhotic or compensated cirrhotic (CC), the probability of experiencing disability in a given year is calculated by adding the risk of experiencing decompensated cirrhosis ($d_{1_{t}}$) to the risk of experiencing HCC ($d_{2_{t}}$) in that year. Patients who are disabled are assumed to be unable to work. As such, the probability of disability is then multiplied by the expected earnings in that year (***ε***_***t***_), estimated using the probit and Poisson models described in Section II.i. Measures of income in household surveys are often far less than reported expenditures (net saving/dissaving) and average income estimates derived from national accounts data. Household surveys likely underestimate income for several reasons: affluent households often refuse to be surveyed or are more difficult to reach, individuals forget or intentionally exclude some sources of income, and individuals choose to underreport income [32,33]. To offset this downward bias and the fact that the IHDS reports income after taxes and other fees, income is adjusted upward using a multiplier (*M*) to approximate its actual pretax value. This represents the full value of the individual’s labor, which exceeds what they take home in earnings. In the standard calculation, *M* equals the ratio of GDP per capita in India in 2016, taken from World Bank [18], to probability-weighted mean earnings reported in the IHDS. (The value of the income multiplier is 5.58 = 114,666 INR/20,550 INR.) This reflects the expectation that the IHDS sample is representative of the Indian population as a whole and thus should reflect the same average income level. Because the GDP per capita figure is for 2016, this multiplier also accounts for the substantial income growth that occurred in India between 2012 (when IHDS wave 2 occurred) and 2016 (when the Nielsen patient survey data were collected). In sensitivity analyses, we also run a conservative estimate that sets the earnings multiplier equal to half of the value of M.

The value of earnings lost to disability is also adjusted to account for time discounting and additional income growth occurring before the benefits accumulate. These benefits are then summed for each year after the survey, up to the year in which the survey respondent would be age 100 if he or she were still living. Because income changes with age, expected earnings are reestimated at each future age using the Poisson regression. The equation for calculating benefits of averted disability, *L*_*dis*_, is provided below:

$$L_{dis}=\sum_{t=1}^{100-a_{0}}{M\varepsilon }_{t}(d_{1_{t}}+d_{2_{t}}){\left(\frac{1+\sigma }{1+\delta }\right)}^{t}$$

Where

*t* = number of years after the individual was surveyed,

*a*_0_ = age of the respondent at the time of interview,

*M* = multiplier to account for underreporting of income,

*ε*_*t*_ = estimated earnings of the respondent at time *t*,

$d_{1_{t}}$, $d_{2_{t}}$ = risk of having decompensated cirrhosis ($d_{1_{t}}$) or HCC ($d_{2_{t}}$) at time *t*,

*σ* = the rate of growth of real income per capita in India, and

*δ* = annual discount rate, 3%.

Figures are calculated under three income growth scenarios. The high scenario assumes that real per capita income growth in India continues at the average rate of the last 5 years, 5.6% annually [18]. The low scenario assumes real per capita income grows at 3% annually, equal to the chosen discount rate. The median scenario is set equal to the midpoint between the high and low scenarios, 4.3%.

Individuals with decompensated cirrhosis are assumed to experience zero benefits of averted disability from HCV treatment. Decompensated cirrhotic individuals generally continue to experience disability after being cured of HCV infection if they do not undergo liver transplantation, which is extremely rare in India.

#### Benefits of averted premature death (L_death_)

The expected risk of death averted in a given year is calculated by adding the chance of having died of liver disease by that point in time (*x*_*t*_) to the chance of dying from other causes (*o*_*t*_) and then subtracting the mortality risk of an individual of the same age and sex but with normal Indian health status (*c*_*t*_). The value of these lost years is monetized, adjusted for growth and discounting, and summed using the same transformations applied to the value of years lived with disability (YLDs) averted:

$$L_{death}=\sum_{t=1}^{100-a_{0}}M\varepsilon_{t}(x_{t}+o_{t}-c_{t}){\left(\frac{1+\sigma }{1+\delta }\right)}^{t}$$

Where

*x*_*t*_ = the probability of having died from HCV-related causes by time *t*,

*o*_*t*_ = the probability of having died from other causes by time *t*, and

*c*_*t*_ = the probability of an individual of the same age and sex, but with average Indian health status, having died from any cause by time *t*.

For decompensated cirrhotic individuals, additional years lived are assigned no value, reflecting the assumption that these years will be spent in disability and without the ability to work.

#### Herd benefits

To calculate the herd benefits (i.e., the benefits to secondarily infected individuals) of curing HCV, the average annual transmission rate of HCV in India (*α*) is multiplied by the likelihood of survival [(1−*x*_*t*_−*o*_*t*_)] to a given year after the time of interview (*t*). Average transmission rate is calculated simply as the estimated number of new HCV infections divided by the estimated total viremic population, as provided in Saraswat et al. [34]. This calculation yields the expected number of individuals the patient would infect in each year after the interview, were they to go untreated.

For each of these years, the expected years of life disabled (YLDs) and years of life lost (YLLs) are calculated for a hypothetical secondarily infected individual in the same region as the primary infected individual. Given that diagnosis is associated with socio-economic status (whereas this is not necessarily the case with infection) it is assumed that the population of HCV *infected* individuals more closely reflects the general Indian population than the HCV *diagnosed* population. This assumption is conservative insofar as medical procedures are a primary vector of transmission. Thus, secondarily infected individuals would likely use some of the same medical facilities as the primary patients and as such might be closer to their socioeconomic status than the average member of the population. Secondarily infected individuals start at stage F0 fibrosis and are assumed to have average population characteristics (as represented in IHDS wave 2) except their age, which is set 20 years younger than the individual who infected them. This approach is intended to create a group of secondarily infected individuals that reflects overall population characteristics but with the age structure of the patient sample. The 20-year age reduction is intended to approximate the substantial time lag that typically occurs between the time of infection and the first diagnosis with HCV. (Two individuals in the sample are age 19. They are assumed to infect newborns.)

The value of the YLDs and YLLs are summed across all years of the secondarily infected individual’s life up to age 100 (at which time the individual has only a small chance of still being alive, irrespective of health status). The lifetime total values of averted YLDs and YLLs are multiplied by the expected number of individuals infected by the patient in a given year. These values are then summed across all years of the primary infected individual’s life. The equations for calculating the herd benefits of averted disability and premature death are provided below:

Benefits of averted disability for secondarily infected individuals (**X**_**dis**_)

$$X_{dis}=\sum_{t=1}^{100-a_{0}}\left[\alpha (1-x_{t}-o_{t})\sum_{i=1}^{100-{(a}_{0}-20)}{M\epsilon }_{i}({d_{1}}_{{F0}_{i}}+{d_{2}}_{{F0}_{i}}){\left(\frac{1+\sigma }{1+\delta }\right)}^{t+i}\right]$$

Benefits of averted premature death for secondarily infected individuals (**X**_**death**_)

$$X_{death}=\sum_{t=1}^{100-a_{0}}\left[\alpha (1-x_{t}-o_{t})\sum_{i=1}^{100-{(a}_{0}-20)}{M\epsilon }_{i}(x_{{F0}_{i}}+o_{{F0}_{i}}-c_{i}){\left(\frac{1+\sigma }{1+\delta }\right)}^{t+i}\right]$$

Where

*i* = the number of years after the secondarily infected individual was infected (starting from fibrosis stage F0),

*ϵ*_*i*_ = the estimated earnings of the secondarily infected individual *i* years after he or she was infected (assuming average population health),

$d_{1({F0}_{i})},\;d_{2({F0}_{i})}$ = risk of having decompensated cirrhosis ($d_{1({F0}_{i})}$) or HCC ($d_{2({F0}_{i})}$) for the secondarily infected individual at time *i*,

$x_{{F0}_{i}}$ = the probability of the secondarily infected individual dying of HCV-related causes by time *i*, and

$o_{{F0}_{i}}$ = the probability of the secondarily infected individual dying of non-HCV-related causes by time *i*.

For individuals who are decompensated cirrhotic at the time of interview, we assume that 29.4% are in their first year of decompensation. Although the time when surveyed individuals first developed decompensated cirrhosis is unknown, the composition of first-years would converge on 29.4% given the relative mortality rates of 0.4 for first-year and 0.25 for non-first-year decompensated cirrhosis [28] if we assume that the number of newly decompensated individuals remained constant each year.

### II.iv. Applying the value of curing HCV to estimate the net value conferred by SOF/VEL treatment

The net value of SOF/VEL is a function of the value of curing HCV infection, the efficacy of treatment, and the cost of treatment.

Treatment guidelines suggest that HCV carriers who fail their first round of treatment with SOF/VEL should generally be administered a second round of treatment [35,36]. Assuming these guidelines are followed, the expected benefits of treatment (*T*) can be calculated by multiplying the value of curing HCV (*C*) determined earlier by the probability of being cured after two rounds of treatment, or

$$T=C[F_{1}+(1-F_{1})F_{2}]$$

where *F*_1_ and *F*_2_ equal the efficacy of the first and second round of treatments, respectively. The efficacy data are the results from ASTRAL-1, ASTRAL-3, and ASTRAL-4 clinical trials. Treatment included 12 weeks of SOF/VEL administration for non-cirrhotic and compensated cirrhotic patients, and 12 weeks of SOF/VEL plus ribavirin for decompensated cirrhotic patients. Although these efficacy figures are used to simulate patient outcomes, efficacy in real-world usage is likely somewhat lower than efficacy in carefully controlled clinical trial conditions.

Table 4 shows treatment efficacy by disease genotype and level of liver functionality. The efficacy data are the results from ASTRAL-1, ASTRAL-3, and ASTRAL-4 clinical trials. Treatment included 12 weeks of SOF/VEL administration for non-cirrhotic and compensated cirrhotic patients, and 12 weeks of SOF/VEL plus ribavirin for decompensated cirrhotic patients. Although these efficacy figures are used to simulate patient outcomes, efficacy in real-world usage is likely somewhat lower than efficacy in carefully controlled clinical trial conditions.

**Table 4:** SOF/VEL Efficacy by round of treatment, disease genotype, and level of liver functionality.

Liver functionality, genotype	F_1_ (%)	F_2_ (%)	Total efficacy (%)
**Non-cirrhotic, G1**	98	100	100.0
**Non-cirrhotic, G3**	98	94	99.9
**Compensated cirrhotic, G1**	100	98	100.0
**Compensated cirrhotic, G3**	93	89	99.2
**Decompensated cirrhotic, G1**	96	96	99.8
**Decompensated cirrhotic, G3**	85	85	97.8
**Weighted average**	**93.9**	**91.9**	**99.3**

**Sources:** [13,14,37].

Table 5 Details the expected cost of the first round of treatment (***P***_**1**_) and second round of treatment (***P***_**2**_). Drug price estimates were taken from Dandekar & Raghavan [15]. All other cost estimates were determined through consultation with Susheel Sule, former Marketing Director for Gilead Sciences, India, and verified by, Professor Abhijit Choudhury, an independent consultant.

**Table 5:** Breakdown of treatment costs (in INR).

	First treatment	Second treatment (i)
**Cost of medication**	52,500	52,500
***Treatment costs at private hospitals and clinics***	** **	** **
HCV antibody test	1,075	0
2 viral count tests (one before treatment and one after treatment) at 5,000 INR each	10,000	5,000
Visits to Gastroenterologist/Consulting Doctors (ii)	5,000	2,000
**Total cost of treatment at private hospitals and clinics**	**68,575**	**59,500**
***Treatment costs at government hospitals*** (iii)		
HCV antibody test	500	0
2 viral count tests (one before, one after treatment) at 2,200 INR each	4,400	2,200
Visits to Gastroenterologist/Consulting Doctors, Provided Free	0	0
**Total cost of treatment at government hospitals**	**57,400**	**54,700**
**Total weighted cost**	***P***_**1**_ = **62,988**	***P***_**2**_ = **57,100**

Notes: (i) The second treatment does not require an HCV antibody test because one has already been performed as part of the first treatment. Similarly, there is no need to perform a viral count test before the second treatment. Only one doctor visit is needed for the second round of treatment because a second prescription would have been written for the patient during the follow-up to the failed first round of treatment. (ii) The visits to consulting doctors include three visits during the first round of treatment: An initial visit where the doctor orders an HCV antibody test followed by a viral count test, a brief consultation (assumed to be half the cost of the other visits) following the initial tests to discuss the test results and the treatment plan, and a follow up after 12 weeks of treatment are completed to discuss the results of the SVR test. If another round of treatment is needed, one additional visit should be held after the second treatment round is completed. (iii) Costs of tests at government hospitals vary widely from facility to facility. The figures in the chart are based on costs in government hospitals in Punjab, which are taken to be representative of the median. (iv) These calculations assume that 50% of treatment occurs in government facilities and 50% occurs in private facilities. Currently 71% of treatment for HCV occurs in private hospitals and clinics. However, it is expected that a higher proportion of treatments will be administered in government facilities as access to treatment expands to rural and semi-urban areas.

Expected treatment value (*T*), treatment costs (*P*_1_ and *P*_2_), and the efficacy of the first round of treatment (*F*_1_) are then used to estimate net value of treatment (*Z*) as follows:

$$Z=T-P_{1}-P_{2}(1-F_{1})$$

## III. Results

Applying the data on sex and liver functionality of the patient population found in the Nielsen Physician Survey (weighted to reflect the physician population of India) suggests that curing the average chronic HCV-carrying patient (i.e., the average individual diagnosed with the disease) would preserve 36,83,290 INR in earnings (see Table 6).

**Table 6:** Expected costs and benefits of SOF/VEL treatment (in INR).

	Non-cirrhotic		Compensated cirrhotic		Decompensated cirrhotic		Total Population
	Male	Female	Male	Female	Male	Female	
**Cure value**	53,97,806	6,62,594	1,06,55,300	16,86,937	14,586	14,854	**36,83,290**
**Expected medication cost (52,500 INR per round)**	53,550	53,550	55,145	55,145	58,757	58,757	**55,707**
**Expected treatment cost**	10,580	10,580	10,719	10,719	11,036	11,036	**10,769**
**Total efficacy (after 2 treatments)**	99.9%	99.9%	99.4%	99.4%	98.3%	98.3%	**99.3%**
**Net benefit**	53,28,278	5,97,801	1,05,25,504	16,10,952	-55,455	-55,192	**35,91,031**
**Expected years until benefits of treatment offset costs**	6	12	1	3	.	.	**3**

Note: Total values are calculated as the average of the values for the different cirrhosis stages and sexes, weighted according to their joint distribution in the Nielsen patient data.

The largest expected treatment benefits are conferred to compensated cirrhotic men (see Tables 7 and 8). Compensated cirrhotic individuals have a high risk of experiencing disability and premature death in the future if they go untreated. However, if cured of HCV infection, they can function as well as other uninfected members of the population. On the other hand, although decompensated cirrhotic individuals face the highest HCV burden, they are unable to fully benefit from treatment as they will continue to experience disability and remain unable to work after clearing infection. Thus, they experience no direct productivity boost from being cured of HCV without further treatment. Treating decompensated cirrhotic individuals also yields relatively small benefits in the form of prevented future infections, because they are likely to live only a few more years if they are treated and thus have a lower chance of transmitting infection in their remaining lifespan.

**Table 7:** Average estimated value (INR) of curing HCV by disease status and sex.

	*Benefits to the individual*	*Benefits of prevented future infections*	*Total benefits*
**Non-cirrhotic males**	51,66,993	2,30,813	53,97,806
**Non-cirrhotic females**	4,74,138	1,88,456	6,62,594
**Compensated cirrhotic males**	1,05,71,850	83,450	1,06,55,300
**Compensated cirrhotic females**	15,98,938	87,999	16,86,937
**Decompensated cirrhotic males**	0	14,586	14,586
**Decompensated cirrhotic females**	0	14,854	14,854
**Weighted average**	35,73,999	1,09,194	36,83,290

**Table 8:** Average estimated value in US dollars of curing HCV by disease status and sex.

	*Benefits to the individual*	*Benefits of prevented future infections*	*Total benefits*
**Non-cirrhotic males**	80,646	3,603	84,249
**Non-cirrhotic females**	7,400	2,941	10,342
**Compensated cirrhotic males**	165,005	1,302	166,307
**Compensated cirrhotic females**	24,956	1,373	26,330
**Decompensated cirrhotic males**	0	228	228
**Decompensated cirrhotic females**	0	232	232
**Weighted average**	55,783	1,635	57,417

Men experience relatively larger productivity benefits than women because of both market and disease dynamics. In the absence of HCV, men’s probability of labor force participation and mean earnings are higher than those of women. Thus, averting disability for men will on average preserve more earnings than it would for women. Progression of HCV-related liver damage is also slower among women than men. Accordingly, a non-cirrhotic, HCV-positive female’s risk of disability and death from HCV is substantially lower than that of a non-cirrhotic, HCV-positive male.

For non-cirrhotic and compensated cirrhotic men and women, individual benefits are much larger than those conferred by preventing future infections. This is partially a result of the patient sample being much more highly educated—and thus having much higher expected earnings—than the Indian population as a whole (compare Tables 1 and 3). For men, this gap is wider because they can infect both men and women, who are on average lower earning than the primary infected males. Women may also pass infection to either males or females, but women’s earnings are lower, making the gap between their direct benefits and herd benefits somewhat smaller.

Treatment with SOF/VEL (which has an expected total cost of 66,476 INR for the average patient) yields net benefits for non-cirrhotic and compensated cirrhotic individuals. While this net benefit is larger for men than for women, the net positives are very large in either case. However, the benefits of averted future infection are not enough to offset the cost of treatment for decompensated cirrhotic men or women. A 12-week supply of medication would need be sold for 3,024 INR (US$ 47) or less in order for the cost of treatment to be offset by the productivity-related herd benefits of treating decompensated cirrhotic individuals.

### Sensitivity analyses

Sensitivity analyses explore the impact of changing assumptions about the income growth the Indian economy is expected to experience in the coming years, the multiplier from reported to actual income, and the cost of the medication. Table 9 presents different assumptions used in the baseline, high, and low estimates and brief explanations of the values selected. Table 10 presents the cure values and net benefits calculated using these different assumptions.

**Table 9:** Assumptions used in sensitivity analyses.

Parameter	Low estimate	Baseline estimate	High estimate
**Average annual growth in real income per capita**	3% (= annual time discount rate)	4.3% (midpoint between high and low estimates)	5.6% (= average growth in real income per capita in India, 2012–2016)
**Multiplier from reported income to actual income**	2.78 (50% of the baseline estimate value)	5.58 (ratio from mean income reported in IHDS wave 2 to GDP per capita reported in WDI)	5.58
**Cost of Medication**	52,500 INR per round of treatment	52,500 INR per round of treatment	7,689 INR per round of treatment

Note: 52,500 INR is the cost of a 12-week course of treatment with generic SOF/VAL taken from Dandekar & Raghavan [15]. 7,689 INR is the price the Punjab government currently pays for a single 12-week course of SOF/VAL medication, which they use for public provision [38]. This number is used in high net value scenario because it is assumed that it may be possible for other states to negotiate the same purchasing price if they choose to provide the drug publicly.

**Table 10:** Cure values and net benefits (INR) under high, low, and baseline assumptions.

	Non-cirrhotic		Compensated cirrhotic		Decompensated cirrhotic		Total Population
	Male	Female	Male	Female	Male	Female	
**Cure Value (Baseline)**	53,97,806	6,62,594	1,06,55,300	16,86,937	14,586	14,854	36,83,290
**Cure value (low estimate)**	18,80,078	2,24,282	41,13,655	6,67,873	4,126	4,189	13,73,101
**Cure value (high estimate)**	79,13,631	10,49,057	1,40,18,857	21,76,387	26,266	26,756	50,50,519
**Net benefit (Baseline)**	53,28,278	5,97,801	1,05,25,504	16,10,952	-55,455	-55,192	35,91,031
**Net benefit (low estimate)**	18,14,068	15,99,28	40,23,109	5,98,003	-65,737	-65,675	12,40,428
**Net benefit (high estimate)**	78,87,295	10,29,585	1,39,15,948	21,44,533	6,178	6,660	50,10,950

Note: Total values are calculated as the average of the values for the different cirrhosis stages and sexes, weighted according to their joint distribution in the Nielsen patient data.

These sensitivity analyses reveal that the costs of treating non-cirrhotic and compensated cirrhotic individuals are dwarfed by the benefits of treatment, even under the most conservative assumptions examined. Under the high value, low cost assumptions, treating decompensated cirrhotic individuals with SOF/VEL is determined to be cost-beneficial on the basis of herd effects alone.

Additional estimates are produced applying the parameters utilized in the baseline specification of Aggarwal et al. [20] (i.e., the discount rate is set equal to zero and the cost of a twelve-week SOF/VEL treatment regimen is assumed to be $300) and the baseline income multiplier (shown in Table 9). Under these assumptions the productivity related benefits of treatment are expected offset the cost of treatment in one year for cirrhotic patients and in five years for non-cirrhotic patients. In comparison, Aggarwal et al. [20] estimate treatment to be cost-saving within five and 12 years for cirrhotic and non-cirrhotic patients, respectively.

### Macroeconomic benefits of treating 10% of the HCV viremic population of India

The average net benefits of SOF/VEL treatment are used to estimate the total economic impact of treating 10% of the HCV-positive population of India (who are assumed to have the same average characteristics as the patient sample with respect to sex and liver functionality). This value is calculated by multiplying the average net benefits of treatment by the treatment population size, 600,000, 10% of 6 million HCV-viremic individuals [4]. Following this approach, the total economic impact is estimated to be 2.2 trillion INR (or US$ 33.7 billion). This value can be considered an upper-bound estimate of the possible macroeconomic effects resulting from the average individual benefits calculated in the baseline scenario. It is an upper bound because it relies on the assumption that the Indian market has a flat demand curve for labor, i.e., that the market will absorb all the additional labor available at the same wage levels and that the additional workers will not crowd out other potential workers.

However, some of the additional healthy adults who would remain in the labor force if treated would likely displace the employment of other workers (who would be healthy in either scenario) or that these additional workers would put downward pressure on wages. In the extreme case, the additional healthy workers would only hold employment positions that would otherwise be filled by other members of the Indian labor force and the net macroeconomic benefits of treatment would be null (or negative if the increased supply of labor reduced equilibrium wages).

The macroeconomic impact of treating 10% of India’s HCV-positive population would fall somewhere between these extreme scenarios. Here it is important to consider that the additional living, healthy, and employed individuals would utilize domestic goods and services. This increased demand for goods and services leads to an increase in demand for labor in India, creating additional employment opportunities. Determining the exact ratio of healthy life years preserved to years of employment added to the Indian economy is a complex macroeconomic modeling task beyond the scope of this paper. Despite these caveats, the upper-bound estimate of this aggregate impact gives an idea of the scope of returns to treatment possible on a national scale.

## IV. Conclusions

The analyses demonstrate that the benefits of increased future productivity for non-cirrhotic and compensated cirrhotic individuals easily justify the cost of treatment with SOF/VEL, even under conservative assumptions about these benefits. Previous research has examined the impact of SOF/VEL treatment on averting future costs of treating late stage symptoms of HCV infection, and shows treatment with SOF/VEL to be cost-saving within five to 12 years [20]. However, we find that the productivity related benefits of SOF/VEL treatment offset the cost of treatment much more quickly than these earlier results suggest—within 20% and 42% of the time previously estimated, depending on the stage of the illness. This result demonstrates the importance of assessing the impacts of medical treatment on productivity, and the extent to which this may encourage greater public investment in health-related interventions.

While the cost of treating decompensated cirrhotic individuals is not offset by gains to future market productivity, this does not imply that these individuals should not receive treatment.

First, these analyses only consider the impact of treating HCV without undergoing liver transplantation. While liver transplantation is prohibitively expensive for the vast majority of Indians, the tremendous gains to extending healthy life expectancy offered by liver transplantation and SOF/VEL together could possibly contribute enough of a productivity boost to offset this cost over the life course.

Another, more important, limitation is that the analyses included in this paper only estimate the additional market productivity preserved by treating HCV infection with SOF/VEL. This is just one component of the benefits of maintaining or restoring health. Curing HCV infection can also help maintain unpaid productive activities, such as caring for children, doing household work, or providing support to other adults. Although HCV is rarely symptomatic in children (because of the time taken for liver function to degrade), the disease’s impact on adults can interfere with their children’s education and development. Shocks to parental health that reduce their earnings may limit the household’s access to nutritious foods necessary for healthy physical and cognitive development or may necessitate that children leave school and work to provide another source of income [39]. As such, current adult HCV infection can obstruct children’s human capital accumulation and thus impose additional economic burden extending decades into the future. These impacts on productivity also have downstream implications for tax revenues collected by the Indian government.

On top of these concrete costs are the aspects of the disease burden that are not as tangibly connected to monetary value: the discomfort, pain, and anxiety caused by the illness and the extent to which it prevents individuals from enjoying leisure and engaging in activities they find fulfilling. The value of treatment also includes the intrinsic value of years of life that would be lost to HCV infection without treatment.

Estimates of the value of HCV treatment that attempt to encompass all of these components by using the statistical value of disability-adjusted life years lost place the value of treatment for decompensated cirrhotic individuals well above the threshold necessary to justify the cost of treatment [20]. However, this approach has some notable disadvantages. The value of statistical life years (VSLY) utilized in these studies is based on observation of tradeoffs that individuals make between mortality risk and income. The values derived from these tradeoffs may not reflect the true value of the life years for several reasons—key among these are individual mis-assessment of risks, general issues with revealed preference studies, and bias introduced in stated preference studies. For these reasons and others, such studies produce a wide range of possible VSLYs [40]. Even supposing the chosen VSLY is accurate, studies utilizing these figures provide no insight on the composition of this value; they do not distinguish between the value individuals separately place on the benefits of increased productivity, effects on children, health gains *per se*, or any other positive results of a given treatment.

Furthermore, neither the approach taken in this paper nor the VSLY-approach account for the distributional impacts of a large-scale program to increase access to SOF/VEL treatment. Providing low-income HCV carriers with access to treatment they otherwise could not afford would address economic inequality by improving lifetime earnings and savings among poor households. Here, the source of funding and characteristics of the treatment recipients would determine the extent to which public provision would be redistributional.

Although this paper’s calculations capture only part of the benefits of treating HCV, the findings are uniquely valuable for quantifying a clearly defined element of this value in an empirically determined manner. These findings have the additional advantage of being tailored to the specific socioeconomic characteristics of the population diagnosed with HCV in India. As such, they offer a compelling argument that HCV treatment will reap very large market returns for most of the national HCV-positive population. These market returns should serve as one of many motivating factors supporting increased access to and utilization of DAA treatment across India.

## Supporting information

S1 File Regression coefficients from IHDS data. (DOCX)
