# Supplementary material for: Estimating the net value of treating hepatitis C virus using sofosbuvir-velpatasvir in India
Source: PLoS One. 2021 Jul 22;16(7):e0252764. doi: 10.1371/journal.pone.0252764 (PMC8297876; doi:10.1371/journal.pone.0252764)
Supplement: S1 File — (DOCX) [file pone.0252764.s001.docx]

**Appendix**

| **Table S1: Regression Coefficients from IHDS Data** | | |
| --- | --- | --- |
| **Dependent variable** | **Employment** | **Earnings** |
| **Model Type** | **Probit** | **Poisson** |
| **Age** | 0.129*** | 0.095*** |
|  | (0.00) | (0.00) |
| **Age^2** | -0.002*** | -0.001*** |
|  | (0.00) | (0.00) |
| **Female** | -1.268*** | -0.643*** |
|  | (0.01) | (0.00) |
| **Married** | 0.168*** | 0.103*** |
|  | (0.01) | (0.00) |
| **Number of household members** | -0.035*** | -0.014*** |
|  | (0.00) | (0.00) |
| **Level of education** |  |  |
|  |  |  |
| **Illiterate** | Base | Base |
|  |  |  |
| **Literate with no formal schooling** | -0.158*** | 0.160*** |
|  | (0.04) | (0.00) |
| **Up to 4 years of schooling** | -0.043** | 0.245*** |
|  | (0.02) | (0.00) |
| **5 to 9 years of schooling** | -0.271*** | 0.464*** |
|  | (0.01) | (0.00) |
| **Secondary School Certificate (SSC) or Higher Secondary School Certificate (HSC)** | -0.562*** | 0.881*** |
|  | (0.01) | (0.00) |
| **Some college or a bachelor's degree** | -0.698*** | 1.410*** |
|  | (0.02) | (0.00) |
| **Above a bachelor's degree** | -0.477*** | 1.744*** |
|  | (0.02) | (0.00) |
| **Constant** | -0.993*** | 8.595*** |
|  | (0.04) | (0.00) |
| **Indian States’ fixed effects** | *Yes* | *Yes* |
| ** p<0.05, ** p<0.01, *** p<0.001* |  |  |
| *Standard errors provided in parentheses* |  |  |
